# P-1039. Plasma Cathelicidin (LL37) Levels Are not Associated with *Candida* Gut Colonization in Critically Ill Patients

**DOI:** 10.1093/ofid/ofae631.1229

**Published:** 2025-01-29

**Authors:** Maya Pimentel, Truc Cecilia Tran, Shiva Murali, Andrea M Detranaltes, Giselle Ortiz, Marissa G Schettino, Abigail A Amaya, Husna Malikzad, Muhammad H Virk, Asmita Ghosh, Roberta Higgins, Shubhra Singh, Kirsten Rydell, Mary N Jones, Rachel Atterstrom, Blake M Hanson, Rodrigo de Paula Baptista, Samuel A Shelburne, Tor Savidge, Cesar A Arias, Max W Adelman

**Affiliations:** Texas A&M University, Houston, Texas; Houston Methodist Research Institute, Houston, TX; Houston Methodist Research Institute and Weill Cornell Medical College, Houston, Texas; Houston Methodist Hospital, Houston, Texas; Houston Methodist Hospital, Houston, Texas; Houston Methodist Hospital, Houston, Texas; Houston Methodist Hospital, Houston, Texas; Houston Methodist Hospital, Houston, Texas; Houston Methodist Hospital, Houston, Texas; Houston Methodist Hospital, Houston, Texas; Houston Methodist Hospital, Houston, Texas; Houston Methodist Hospital, Houston, Texas; Houston Methodist Hospital, Houston, Texas; Houston Methodist Hospital, Houston, Texas; Houston Methodist Hospital, Houston, Texas; The University of Texas Health Science Center, Houston, Texas; Houston Methodist Hospital, Houston, Texas; MD Anderson-University of Texas, Houston,, Texas; Baylor College of Medicine, Houston, Texas; Houston Methodist and Weill Cornell Medical College, Houston, TX; Houston Methodist Hospital, Houston, Texas

## Abstract

**Background:**

In the intensive care unit (ICU), *Candida* spp. gut colonization is a risk factor for *Candida* bloodstream infection; however, the specific risk factors for *Candida* gut colonization remain unknown. Impaired anti-microbial immune responses may predispose to *Candida* gut colonization. LL37, an antimicrobial peptide (AMP) from the cathelicidin family, plays an important role in the immune response to pathogens, including *Candida*. The purpose of this study was to determine whether plasma LL37 levels are associated with *Candida* gut colonization in the ICU.

**Figure 1.**
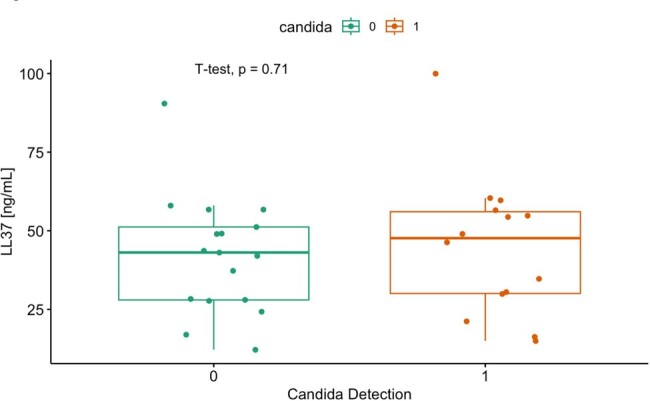
Plasma LL-37 levels comparing patients with (Candida=1) and without (Candida=0) Candida gut colonization.

**Methods:**

We examined a sample of patients enrolled in DYNAMITE (a prospective cohort study of ICU patients at a tertiary care hospital) who provided stool samples. Clinical data was collected by chart review. The first stool sample collected after enrollment was diluted in saline and plated on selective media for *Candida* spp.; species was identified from positive samples via matrix-assisted laser desorption/time-of-flight. Plasma samples were analyzed for LL37 levels via enzyme-linked immunosorbent assay. LL37 levels were normalized with a four-parameter logistic curve. We compared plasma LL37 levels between patients with and without *Candida* gut colonization, as well as different colonizing *Candida* species, using an unpaired *t*-test in R.

**Figure 2.**
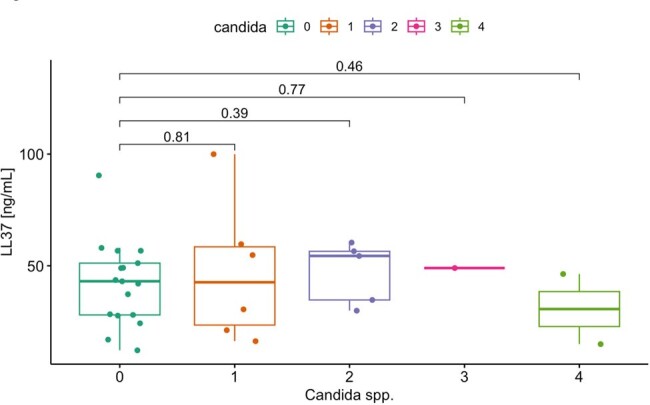
Plasma LL-37 levels comparing patients with different Candida species causing gut colonization (0=no Candida gut colonization; 1=C. albicans; 2=C. glabrata; 3=C. parapsilosis; 4=C. albicans/C. glabrata co-colonization).

**Results:**

Of 31 patients included, 14 (48%) had *Candida* gut colonization (6 [19%] *C. albicans*; 5 [16%] *C. glabrata*; 2 [6%] *C. glabrata* and *C. albicans*; 1 [3%] *C. parapsilosis*). Mean age was 60±16 years; 22 (71%) patients were white; 16 (52%) were women; and 10 (32%) were in shock on ICU admission. There was no statistically significant difference in plasma LL37 between patients with and without *Candida* gut colonization (mean 44.9± 22.6 ng/mL vs. mean 42.0 ±18.8 ng/mL, respectively, p=0.71) (**Figure 1**). LL37 levels also did not differ according to colonizing *Candida* species (**Figure 2**).

**Conclusion:**

In this pilot study, plasma LL37 was not found to be associated with *Candida* gut colonization among ICU patients. These findings suggest that systemic levels may not reflect tissue (intestinal) immune responses. Understanding the role of LL37 and other AMPs in *Candida* colonization may inspire alternative approaches to reduce *Candida* colonization and resultant *Candida* bloodstream infections in the ICU.

**Disclosures:**

**All Authors**: No reported disclosures

